# *Rhodnius prolixus* impairs *Trypanosoma cruzi* growth through cold-seeking behavioral thermoregulation

**DOI:** 10.1371/journal.pntd.0013811

**Published:** 2025-12-08

**Authors:** Henri Loshouarn, Alessandra A. Guarneri

**Affiliations:** 1 Vector Behavior and Pathogen Interaction Group, Instituto René Rachou, Fundação Oswaldo Cruz-FIOCRUZ, Belo Horizonte, Brazil; 2 Univ. Lille, CNRS, Inserm, CHU Lille, Institut Pasteur de Lille, U1019 - UMR 9017 CIIL Center for Infection and Immunity of Lille, Lille, France; Tulane University School of Public Health and Tropical Medicine, UNITED STATES OF AMERICA

## Abstract

Chagas disease, caused by the parasite *Trypanosoma cruzi*, is a major neglected tropical disease affecting 6–7 million people worldwide. *Rhodnius prolixus*, one of the most important vectors of Chagas disease in Latin America, is known to be highly sensitive to environmental temperature, which influences both its biology and parasite development. However, few studies have investigated how this vector behaviorally modulates the effects of temperature by shifting their thermopreference, particularly in response to infection. We investigated how *T. cruzi* infection of *R. prolixus* fourth-instar nymphs influences thermopreference along a temperature gradient, while examining differences across times of day and time since blood feeding. Additionally, parasite load and infection maintenance were compared between free-moving nymphs and nymphs kept at a constant 26°C. Infected nymphs exhibited a preference for temperatures approximately 1°C cooler than uninfected controls. This cold-seeking behavior emerged around 15 days post-infection and persisted until shortly after molting. Importantly, infected insects allowed to thermoregulate showed significantly lower intestinal parasite loads and a higher rate of infection clearance compared to those kept at a constant 26°C. A diurnal cycle in temperature preference was also observed, with higher preferred temperatures shortly after the beginning of the photophase, followed by a gradual decline over the day and night. These results suggest the existence of an infection-induced behavioral anapyrexia response in *R. prolixus* that limits *T. cruzi* development. This potential form of adaptive thermoregulation has important implications for the ecology of Chagas disease transmission and the development of behaviorally informed vector control strategies.

## Author summary

Chagas disease affects millions of people across Latin America and is caused by a parasite transmitted by blood-feeding insects known as kissing bugs. The way these insects interact with temperature is important as temperature influences both their biology and the growth of the parasite. In order to understand whether kissing bugs change their preferred temperature when they are infected, and how this affects the parasite inside them, we allowed infected and uninfected insects to choose their preferred temperature along a gradient. We found that infected insects shifted their preference to slightly cooler temperatures compared to uninfected insects. This cold-seeking behavior began about two weeks after infection and continued until the insects molted. Importantly, insects that were free to seek cooler temperatures had fewer parasites in their gut and were more likely to clear the infection than insects kept at a constant warm temperature.

These findings suggest that kissing bugs may use temperature choice as a defense strategy, which could influence parasite transmission and disease dynamics.

## Introduction

Triatomine bugs (Hemiptera: Reduviidae: Triatominae) comprise more than 150 species of blood-feeding insects distributed across the Americas, all of which are potential vectors of the protozoan *Trypanosoma cruzi*, the causative agent of Chagas disease, although vector competence varies between species [1–3]. *Rhodnius prolixus* is the principal vector species involved in the domestic transmission of this disease in the Andean Community region [4–6]. This parasitic infection occurs primarily within Latin American regions where it represents a significant public health challenge [7]. *Trypanosoma cruzi* is an obligatory parasite that infects both mammals and triatomines, which can function as host reservoirs and/or vectors for the parasite. *Trypanosoma cruzi* transmission usually occurs through contact with the insect vector, emphasizing the importance of understanding the ecological and biological factors influencing *R. prolixus* and its relationship with *T. cruzi* [8].

The environmental temperature that the insect is exposed to is an important factor influencing the physiology, behavior, development, immunity and metabolic rates of the triatomine vector [9–14]. The presence of infection-related effects, including elevated mortality and delayed molting, also depends on the ambient temperature [10,15,16]. Furthermore, *T. cruzi* development, multiplication rate, and first appearance in the feces of the vector are made possible and accelerated by higher temperatures, while colder temperatures decreases the multiplication rate of the parasite [10,15,17–20]. Deciphering the complex and paradoxical effects of temperature is particularly relevant because triatomine distribution and shelter choice have been shown to be tightly linked with temperature [6,21,22], and global warming has been established as the most significant climate change factor affecting insect species [23–25]. These observations rise questions as to whether either the vector or the parasite modulate host temperature preference as part of their interaction.

Indeed, *R. prolixus* plays an active role in mediating the effects of temperature through its thermopreference and its ability to select shelters and oviposition sites based on thermal cues [21]. Adults of *R. prolixus* prefer a temperature between 25–25,5°C on average, and this temperature preference is affected by a range of factors, including sex, with females preferring slightly higher temperatures than males, and nutritional status with the preferred temperature diminishing with the time since the last blood meal. Additionally, diurnal variations in thermopreference have been described, as *R. prolixus* adults display a preference for warmer temperatures at the onset of the scotophase, and for cooler temperatures in the photophase [21]. However, in *R. prolixus*, thermopreference has only been described in adults, leading to interrogations about possible differences between developmental stages.

Crucially, the effect of *T. cruzi* infection on the thermopreference of *R. prolixus* remains unexplored, although this knowledge is fundamental to determine whether infected insects exhibit adaptative behavioral strategies as a defense against the parasite, and the implications of such a behavior for Chagas disease epidemiology are indisputable. Indeed, the applicability of any discovered temperature-related effects on insect biology is reliant on the insect actively seeking and maintaining itself within the identified temperature range, whether it be because it represents the preferred temperature range of the insect or represents the most favorable option available to it.

In this study we aimed at understanding the effects of infection by *T. cruzi* on the thermopreference of *R. prolixus*. Additionally, we compared the molting time and the evolution of parasite load between insects allowed to select their environmental temperature and individuals maintained at a fixed temperature, providing insights into the interaction between behavioral thermoregulation and infection dynamics. We also characterized the variation in thermopreference throughout the day and over the period from blood feeding up to two weeks after molting. We hypothesize that *T. cruzi* infected insects will generally prefer higher or lower temperatures than their uninfected counterparts, potentially as an adaptative defense mechanism against infection. The diurnal variation of thermopreference might also be different between the nymphs, studied here, and the adults studied previously [21].

## Materials and methods

### 1. Ethics statement

All experiments using live animals were performed in accordance with the Fundação Oswaldo Cruz (FIOCRUZ) guidelines on animal experimentation and were approved by the Ethics Committee in Animal Experimentation (Comissão de Ética no Uso de Animais de Laboratório; CEUA/FIOCRUZ) under the approved protocol number LW 03/22. The protocol we used is from the Conselho Nacional de Controle de Experimentação Animal of the Ministério da Ciência, Tecnologia e Inovações (CONCEA/MCTI; http://www.cobea.org.br/), which is associated with the American Association for Animal Science (AAAS), the Federation of European Laboratory Animal Science Associations (FELASA), the International Council for Animal Science (ICLAS) and the Association for Assessment and Accreditation of Laboratory Animal Care International (AAALAC).

### 2. Organisms

The *R. prolixus* colony used in this study originated from insects collected in Honduras in the 1990s. Insects were maintained by the Vector Behavior and Pathogen Interaction Group at the Instituto René Rachou. The colony is maintained at 27 ± 1ºC, 60 ± 5% RH and 12:12 light-dark (LD), and fed monthly on heparinized ram blood. Experimental triatomines were fed on SWR/J mice anesthetized with intraperitoneal injections of a mixture of ketamine (150 mg/kg mg/kg; Cristália, Brazil) and xylazine (10 mg/kg; Bayer, Brazil).

Trypanosome infection was performed using the *T. cruzi* strain Dm28c originally isolated from a naturally-infected *Didelphis marsupialis* [26]. Parasites were cultured *in vitro* by two weekly passages in liver-infusion tryptose (LIT) medium supplemented with 15% fetal bovine serum (FBS), 100 mg/ml streptomycin and 100 UI/ml penicillin [27]. To prevent loss of infectivity, parasites were subjected to cycles of triatomine-mouse transmission every fifteen weeks.

### 3. Infection

The following protocol was repeated five times, for a total of n = 20 insects in each of the “thermal choice groups”, and n = 46 in the “fixed 26ºC infected group”. Two SWR/J mice were infected through intraperitoneal inoculation with 200 μl of triatomine urine containing metacyclic trypomastigotes of *T. cruzi*. On the ninth day after inoculation, the mice were anesthetized and exposed to fourth instar nymphs of *R. prolixus* that molted 75 days prior. This fasting period was chosen to standardize their physiological state and ensure complete metabolization of the last blood meal. Using fourth instar nymphs allows for the observation of thermopreference in the fourth and fifth instars after molting, as these are points in the life cycle at which earlier studies suggest that temperature have the strongest effects, and avoids the adult stage, in which thermopreference has already been described [10,21,28]. The nymphs were allowed to feed until fully engorged. The same protocol was simultaneously conducted with uninfected mice to establish the control group.

Among the individuals that fed to completion, four infected and four controls were randomly selected for the thermopreference experiment, forming the “thermal choice infected” and “thermal choice control” groups. The remaining nymphs that fed on the infected mice were kept individually in plastic containers (4 cm diameter × 2 cm high) covered by cloth, and placed in a climate chamber maintained at 26 ± 0.6ºC, 45 ± 5% RH and 12:12 light-dark (LD) illumination cycle. These nymphs formed the “fixed 26ºC infected” group.

### 4. Experimental procedures

Immediately after feeding, the individuals from the thermal choice groups were placed in the thermopreference apparatus, which consisted of a 54,5 cm long, 24 cm wide and 7 cm high glass arena attached to a thermal gradient system by means of thermo-conductive silicone grease. The arena was divided longitudinally into eight 2,5 cm-wide corridors, each housing one *R. prolixus* nymph for the duration of the experiment. A glass cover was placed on top of the arena to prevent air current interference. The floor of the arena was covered with filter paper, and the walls were coated with opaque plastic to prevent visual interaction between individuals. The thermal gradient was established using a 70 x 25 cm aluminum sheet, heated at one end to 31 ºC with electric heating resistance and cooled at the other end to 18 ºC using a freezer plate. The temperature of each corridor was recorded every 6 cm along the gradient with a thermometer (Hanna HI 2221) prior to the experiment, and ranged from 30.4 ± 0.3ºC at the hottest end to the 20.6 ± 0.3ºC at the coldest. The apparatus was placed in a cupboard under a 12:12 light-dark (LD) illumination cycle, with the scotophase starting at 20:00 and the photophase at 8:00.

The position of the nymphs on the gradient was recorded using a camera (Intelbras IM3 C) placed 48 cm above the arena. A polynomial regression model was fitted in R [29] to the temperatures measured at each point of the corridors. The temperature at which the nymphs were located was calculated by replacing the x in the polynomial by their distance to the hottest end. This distance was determined by analysis of the video recordings in Fiji [30]. The thermopreference was assessed at six different times of day, namely the midpoints of the scotophase and photophase (i.e., 00:00 and 12:00), and two time points bracketing each phase transition (i.e., 07:30, 08:30, 19:30, and 20:30). Thermal choice and fixed 26°C groups were inspected daily for molting and survival throughout the experiment, which spanned from the blood meal to 15 days after 50% of the individuals in the gradient had molted.

At the end of the experiment, the individuals from the infected thermal choice and the fixed 26ºC groups were dissected and their whole intestines were suspended in PBS and macerated. Next, 10 μL of this solution was loaded in a Neubauer chamber for determination of the parasite concentration under light microscopy at 200x magnification.

### 5. Statistical analysis

We used the lme4 package [31] in R (version 4.4.2) [29] to perform generalized linear mixed-effects models (GLMM) analysis of the thermopreference of the insects throughout the experiment. The response variable in these models was the temperature chosen by the insect, and the effect of three dependent variables was studied, namely (i) the number of days since the beginning of the experiment, (ii) the time of day, and (iii) the infection status (i.e., infected or control). The random effect was specified as the individual triatomine sample nested within the day at which each measurement is made. This ensures that the model accounts for the repeated measurement of the same individuals.

Five GLMM models assuming all available fitting data distributions were tested. The best of all models generated was selected based on the lowest Akaike information criterion (AIC). Through this method, the data distribution assumed by the models for the thermopreference of the insects was the inverse Gamma. Each time of day studied was then analyzed separately by creating GLMM models using only data from these times of day. This allowed us to study the effects of the days since the beginning of the experiment and infection independently for each time of day.

The differences in parasite concentrations and time to molt between treatments were tested using the Wilcoxon rank-sum test. Differences in the proportion of insects with undetectable parasites in the intestines between groups were assessed using exact binomial tests, with the presence of live parasites in the sample defined as the success term.

Each graphical representation presented in this article was generated using the ggplot2 package [32]. All numerical values presented in the text alongside confidence intervals represent the means ± standard errors (SEs). In all the statistical tests performed in this study, the level of significance was set to α ≤ 0.05.

## Results

### 1. Temperature preference

The individuals from the *T. cruzi* infected group preferred significantly lower temperatures compared to those from the uninfected control group (t-value = 5.49, p = 3.98e-8, n = 40), on average preferring a temperature of 22.39 ± 0.05 ºC compared to 23.40 ± 0.07 ºC for the uninfected control group when pooling all data from each day and time of day. When analyzed separately for each time of day using individual models, this difference in thermopreference remained statistically significant at nearly all time points (at: 00:00, t-value = 4.73 and p = 2.28e-6; 07:30, t-value = 5.32 and p = 1.04e-7; 12:00, t-value = 4.95 and p = 7.62e-7; 19:30, t-value = 5.43 and p = 5.81e-8; 20:30, t-value = 5.13 and p = 2.87e-7), with the exception of 08:30 where no significant difference in thermopreference was detected between the infected treatment and uninfected control group.

**Fig 1:**
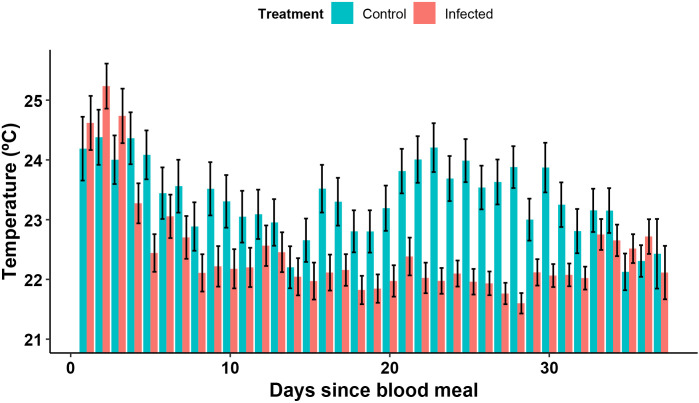
Daily mean preferred temperature of *T. cruzi* infected (red) or uninfected controls (blue) fourth instar nymphs of *R. prolixus* for each day after blood feeding. The preferred temperature was significantly lower in infected insects compared to uninfected controls (t-value = 5.49, p = 3.98e-8, n = 40), and varied significantly across days (t-value = 6.31, p = 2.79e-10, n = 40) based on GLMM analysis. For readability, data from all times of day were pooled within each day. Error bars represent the standard error of the mean.

The thermopreference was different between days (t-value = 6.31, p = 2.79e-10), diminishing in the first 14 days for both treatments (i.e., *T. cruzi* infected and uninfected controls), before stabilizing around 22ºC for the *T. cruzi* infected group, while the control group prefers a higher temperature of around 24 ºC from the 15^th^ day to the 35^th^ day after blood feeding. This effect was not observed in all times of day, as the days since the beginning of the experiment only has a significative effect at 19:30 (t-value = 2.91, p = 3.63e-3), 20:30 (t-value = 2.03, p = 4.2e-2), and 08:30 (t-value = 6.1, p = 1.05e-9), but not at 07:30, 12:00 and 00:00 (Fig 1).

The time of day also influenced the thermopreference of the insects, as highest preferred temperature was observed at 08:30, where it was 23.35 ± 0.11 ºC, which was significantly higher than at any other time of day for both the uninfected and the infected group (difference with 00:00, t-value = -9.04 and p < 2e-16; 07:30, t-value = -9.83 and p < 2e-16; 12:00, t-value = 6.11 and p = 1.02e-9; 19:30, t-value = 5.35 and p = 8.65e-8, 20:30, t-value = 6.88 and p = 5.87e-12).

**Fig 2:**
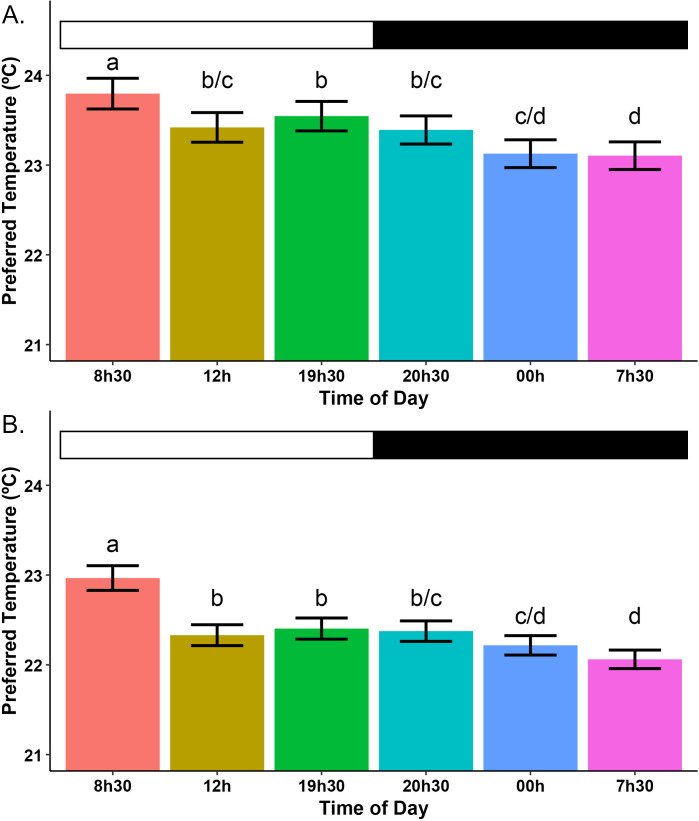
Mean preferred temperature of *R. prolixus* for uninfected controls (A) and *T. cruzi* infected nymphs (B) at different times of day. Data from all experimental days were pooled for each time of day for data visualization. Differences in preferred temperature between times of day and infection treatments were analyzed through GLMM. The preferred temperature of the *T. cruzi* infected group was significantly lower than that of the control group at all times of day except at 8h30, where no significant difference was observed between treatments. Letters indicate statistical groupings between times of day. Black and white bars represent the scotophase and photophase, respectively. Error bars represent the standard error of the mean, and n = 40.

The second-highest preferred temperature occurred at 19:30 (22.9 ± 0.1ºC), which was significantly higher than at 00:00 (t-value = -3.75 and p = 1.75e-4), 07:30 (t-value = -4.61 and p = 4.05e-6), and 20:30 (t-value = 2.03 and p = 4.26e-2), but not significantly different from 12:00. The thermopreference at 12:00 (22.8 ± 0.1ºC) was not different from that at 20:30 but was significantly higher than at 00:00 (t-value = -2.88 and p = 4.03e-3) and 07:30 (t-value = -3.76 and p = 1.71e-4). Similarly, the preferred temperature at 20:30 (22.8 ± 0.1ºC) was higher than at 00:00 (t-value = -2.13 and p = 3.36e-2) and 07:30 (t-value = -3.05 and p = 2.3e-3). The lowest preferred temperatures were observed at 07:30, where it reached 22.52 ± 0.07 ºC, and 00:00 (22.6 ± 0.09ºC), with no significant difference between these two times (Fig 2).

### 2. Infection and molting

**Fig 3:**
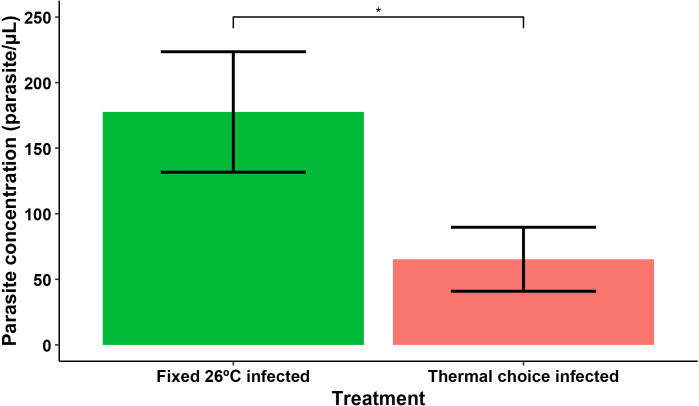
*T.* *cruzi* parasite concentration in macerated whole intestines of fifth instar nymphs of *R.* *prolixus* in fixed 26ºC and thermal choice treatments. Individuals from the thermal choice treatment group exhibited significantly lower parasite concentrations at the end of the experiment compared to those in the fixed 26°C group (Wilcoxon rank-sum test: W = 166.5, p-value = 4.1e-2, n = 34). Error bars represent the standard error of the mean.

The proportion of nymphs with no detectable *T. cruzi* infection by the end of the experiment (i.e., that exhibited no detectable parasite in their macerated intestines under light microscopy) was significantly higher in the free choice infected group when compared to the fixed 26ºC treatment group (Exact binomial test: p-value = 9.59e-4, n = 43). In the free choice group, only 9 of the 16 insects tested exhibited detectable parasites in their intestines (43,8%), compared to 24 out of 27 insects (88,9%) in the fixed 26ºC group. Furthermore, among individuals with detectable infections, the parasite concentration in the intestine was lower in the insects from the thermal choice treatment when compared to the fixed 26ºC treatment group (Wilcoxon rank-sum test: W = 166.5, p-value = 4.1e-2, n = 34) (Fig 3).

**Fig 4:**
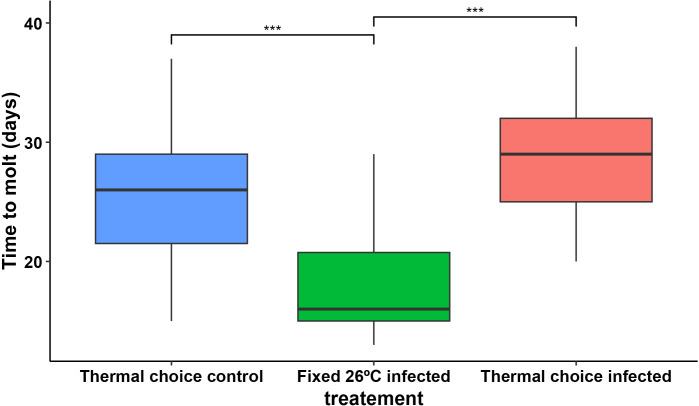
The time from alimentation to molting of fourth instar nymphs of *R. prolixus* across experimental treatments. Nymphs from both thermal choice groups (uninfected and infected) took significantly longer to molt compared to nymphs from the fixed 26°C infected treatment. Statistical comparisons: fixed 26°C infected vs. uninfected thermal choice (Wilcoxon rank-sum test: W = 589.5, p-value = 4.01e-5, n = 61); fixed 26°C infected vs. infected thermal choice (Wilcoxon rank-sum test: W = 42, p-value = 2.56e-8, n = 64).

The time from blood meal to molting was different between treatments, as the insects from the fixed 26ºC treatment group molted in 17,9 ± 0,6 days, which was significantly faster than the insects from both the thermal choice control treatment (W = 589.5, p-value = 4.01e-5, n = 61) and the thermal choice infected treatment (W = 42, p-value = 2.56e-8, n = 64), which took 25.6 ± 1.6 days and 28,7 ± 1,2 days to molt, respectively, with no significant difference between the thermal choice groups (Fig 4).

## Discussion

The thermopreference of fourth instar nymphs of *R. prolixus* recently infected by *T. cruzi* differed significantly from that of uninfected nymphs, in that the infected insects tended to prefer temperatures approximately 1ºC lower on average when compared to their uninfected counterparts. The onset of this behavioral shift emerged around the 15^th^ day after the infective blood meal and persisted until approximately the 34^th^ day, a few days after molting to the fifth nymphal instar. Notably, when compared with *T. cruzi* infected nymphs placed at a fixed temperature of 26ºC, the free choosing group exhibited significantly lower intestinal concentrations of parasites, and a higher proportion seemingly lost infection, with no detectable parasites in the intestines at the end of the experiment compared to those maintained at a constant temperature of 26°C.

Alongside immunological responses, thermoregulation may play an important role in insect defenses against pathogens. Several examples of insects seeking higher temperatures when infected have been described, a behavior known as behavioral fever [33–35], which serves to place the parasite in a thermally suboptimal environment, impairing its multiplication and capacity to chronically infect the host. Conversely, some insects favor cooler temperatures when infected, in order to slow the multiplication of the pathogen, a reaction referred to as “behavioral anapyrexia” or “behavioral chill” [36–39]. Interestingly, *R. prolixus* has been shown to exhibit behavioral fever when inoculated intracelomically with *T. cruzi* [40]. However, as *T. cruzi* does not naturally invade the hemocoel, this response may represent an unspecific reaction to the presence of foreign cells in the hemocoel, suggesting that *R. prolixus* may exhibit pathogen-specific thermoregulatory behaviors, as described in other insect species [33]. The cooler temperature preference observed in our study could thus represent a specific adaptive response aimed at slowing the multiplication of *T. cruzi*, mitigating its effects on the host, and/or enhancing immune efficacy.

Building on this idea, behavioral anapyrexia might emerge, amongst other scenarios, in host species that cannot tolerate the high temperatures required to significantly impair relatively warm temperature-resistant pathogens without suffering detrimental effects that exceed the costs of infection. Indeed, we know that *T. cruzi* can survive and develop at temperatures up to at least 37°C, as evidenced by its ability to thrive in human hosts, although differences in thermal ranges may exist between the different life stages of the parasite. Conversely, previous work from our group show that *R. prolixus* experiences higher mortality and harmful effects at temperatures above 30 ºC [10,13,28,41], potentially rendering it unable to harm *T. cruzi* through behavioral fever. This constraint may explain the emergence of behavioral anapyrexia in *R. prolixus*, as the multiplication of *T. cruzi* is reduced at lower temperatures [10,15], and these temperatures may fall in ranges that induce fewer costs to *R. prolixus*. This interpretation is supported by our earlier findings, in which *T. cruzi* growth rate was lowest at cooler temperatures, while *R. prolixus* survival and water retention rates were at their highest [10,28].

Interestingly, the time of day at which no difference in preferred temperature was observed between infected and uninfected treatment groups was 8:30. This time of day also corresponded to the highest preferred temperature across the six time points examined in this study. Given that this measurement was taken thirty minutes after the onset of the photophase, it could represent a startle response of the insect, or an investigative phase during which the insects would explore the test arena at the beginning of the day, potentially in an attempt to find shelter from the light. The first hour after the onset of the photophase has been observed to be one of the two times of day, along with the onset of the scotophase, at which *R. prolixus* engages in the most spontaneous locomotion even in the absence of a temperature gradient, supporting the hypothesis that this result represents a period of increased locomotory activity, rather than a preference for higher temperatures at 8:30 [42,43].

Regardless of infection status, the insects generally preferred cooler temperatures during the night (00:00 and 7:30) compared to the day (8:30, 12:00, 19:30). At the beginning of the night (20:30), however, the preferred temperature was more similar to daytime levels than to those observed later in the night. This behavior has been described in adult *R. prolixus* and fourth instar nymphs of *Triatoma brasiliensis* [12,21] where this time of day has been associated with the host seeking period, although in our experiment this behavior is expressed even in fed insects that do not require a blood meal. In adult *R. prolixus*, studies suggest that preferred temperatures are higher during the whole night than during the day, which contrasts with our findings at 00:00 and 7:30. However, our results on *R. prolixus* align with observations of fourth instar nymphs of *T. brasiliensis*, which exhibit a preference for warmer temperatures during the day compared to the night. This discrepancy may indicate that the thermopreference patterns of triatomine nymphs differ significantly from those of adults, highlighting potential behavioral differences between developmental stages.

In this study, we demonstrated that *R. prolixus* exhibits behavioral anapyrexia when infected by *T. cruzi*, enabling the insect to impair parasite development, sometimes to the point where the parasite becomes undetectable in the intestine of the triatomine. To the best of our knowledge, this represents the first evidence of behavioral anapyrexia in a major vector of a human parasitic disease, and could have important implications for the future of Chagas disease epidemiology and control, particularly in a warming climate. This cold-seeking behavior in infected vectors may lead them to avoid warmer human dwellings, as urban areas are generally warmer than surrounding natural environments [44], although the motivation of infected vectors to seek cooler environments and the distances they might travel to do so remain unknown.

Alternatively, infected vectors may aggregate in cooler microhabitats that are also prone to human occupation, such as areas near water bodies, potentially increasing transmission risk in those locations. Behavioral chill could complicate efforts to predict disease hotspots, as traditional ecological models often assume static vector behavior and equivalence of behavior between infected and uninfected vectors. However, knowledge of this behavior might help to focus control efforts on infected insects, by increasing trapping and insecticide applications to the right microhabitats, using behavioral insights to develop biologically informed control strategies. To confirm the broader applicability of these findings, field studies involving wild populations of *R. prolixus* are warranted. These studies would also help to overcome the inherent limitations brought by long-term laboratory colonies, such as reduced genetic diversity and altered life-cycle dynamics, potentially influencing behavioral and physiological responses. Overall, our findings highlight the complexity of the thermal behavior of *R. prolixus*, and underscore the importance of understanding the interplay between infection, host behavior, and pathogen development in the context of vector biology.

## Supporting information

S1 File Dataset. (XLSX)

S2 File Dataset. (XLSX)
